# Pentraxin-3 and inflammatory biomarkers related to posterolateral thoracotomy in Thoracic Surgery

**DOI:** 10.12669/pjms.35.2.181

**Published:** 2019

**Authors:** Isa Dongel, Aysegul Aksoy Gokmen, Ibak Gonen, Selcuk Kaya

**Affiliations:** 1 *Isa Dongel, Department of Thoracic Surgery, Faculty of Medicine, Suleyman Demirel University, Isparta, Turkey*; 2 *Aysegul Aksoy Gokmen, Department of Medical Microbiology, Faculty of Medicine, Katip Celebi University, Izmir, Turkey*; 3 *Ibak Gonen, Department of Infectious Disease, Faculty of Medicine, Medical Patk Siliviri Hospital, Istanbul, Turkey*; 4 *Selcuk Kaya, Department of Medical Microbiology, Faculty of Medicine, Katip Celebi University, Izmir, Turkey*

**Keywords:** Pentraxin-3, C-reactive protein, Inflammatory biomarkers, Thoracotomy

## Abstract

**Objective:**

Posterolateral thoracotomy is the most frequently used operation in thoracic surgery, and may initiate an inflammatory process. We aimed to evaluate inflammatory response of the body to posterolateral thoracotomy.

**Methods:**

This study was conducted between January 2013 and June 2014. Blood samples were drawn from 36 patients who underwent posterolateral thoracotomy preoperatively, and on postoperative days one, three and seven The levels of PTX-3, CRP and WBC in the serums of the patients were identified. All the results were recorded and analyzed.

**Results:**

PTX-3 levels were found statistically significantly higher in patients with lung cancer and/or aged above 65 years. There were significant differences in WBC and CRP levels between preoperative levels and on those on postoperative days one, three and seven but not for PTX-3. The area under the curve(AUC) levels in the receiver operating characteristics(ROC) analysis, which was performed to estimate the strength of PTX-3 in the differentiation of malignant and benign patients was found statistically significant(p<0.05).

**Conclusions:**

The present study suggests that the novel inflammatory marker PTX-3 may be used in the diagnosis and follow-up of prognosis as a biomarker of inflammatory response in patients with lung cancer. However, it showed that PTX-3 levels are insignificant to identify the levels of inflamatuar response due to posterolateral thoracotomy in thoracic surgery.

## INTRODUCTION

Posterolateral thoracotomy is the most frequently used operation in thoracic surgery. The patient is positioned in the lateral decubitus position under general anesthesia to enable the surgery region on the upper side, the surgical procedure is performed by expanding the intercostal space after placing a pillow under the chest. Independent of the surgery, posterolateral thoracotomy in itself initiates the inflammatory process in the body. Pentraxin-3 (PTX-3) is a novel acute-phase protein detected as an inflammatory response of the body.^1^-^3^ The pentraxin family is divided into two groups as short, and long; PTX-3 is a prototype of the long pentraxin family (molecular weight, 440 kDa), which is produced by the vascular epithelium, smooth muscle, lung epithelial cells, monocytes, macrophages, fatty tissue, and mostly by the liver in various organs as an inflammatory response of the body.^4^-^7^ The production of PTX-3 is increased by the different proinflammatory cytokines [e.g., interleukin -1b, tumor necrosis factor -α], tissue damage or the stimulation of microbial agents.^8^,^9^

C-reactive protein (CRP) is an acute-phase protein that increases remarkably during infection, inflammation, and with tissue damage. It is mainly produced and secreted into the circulation by the liver in response to circulatory inflammatory mediators. CRP levels are the best indicator to determine tissue damage because CRP increases immediately in the early phase of tissue deterioration.^10^-^12^

In the present study, we aimed to identify changes in the inflammatory biomarkers PTX-3, CRP, and WBC in patients who underwent posterolateral thoracotomy between preoperative levels, and those on postoperative days 1, 3, and 7, and to determine correlations between them.

## METHODS

The present study was planned as a prospective study in the department of Thoracic Surgery in Suleyman Demirel University (SDU), Medical Faculty, with the approval of the local ethics committee of the SDU clinical studies. Post-operative period, patients with infection symptoms and/or fever were excluded from the study. The age, sex, and diagnoses of 36 patients who underwent posterolateral thoracotomy (approximately 20 centimeters) between January 2013 and June 2014 were recorded. The blood was drawn, and PTX-3 (ng/mL), CRP (µg/mL), and WBC levels were studied in the serums preoperatively, and on postoperative days one, three and seven. Approximately 3 mL blood was drawn to identify the PTX-3 levels, and the blood was centrifuged at 2000 *g* in the microbiology laboratory to purify from the shaped components, the remaining serum was placed in Eppendorf tubes and stored at -80°C. The study was completed approximately in 18 months. All of the values of preoperative and postoperative days one, three and seven PTX-3, CRP, and WBC levels in patients serum were quantitatively studied in a double-blind fashion in serums of patients using microelisa Human-PTX-3 ELISA Kit (Boster Biological Technology, CA) in the microbiology laboratory of Katip Celebi University Medical Faculty, in accordance with the protocol and standards and read using an ELISA plate reader at 450 nm wavelength (Bio-Tek ELx808, USA).

### Statistical Analysis

All results were recorded and analyzed using the Statistical Package for the Social Sciences (SPSS) Ver. 22 program. The statistical analysis was performed with 95% confidence intervals. Bonferroni-corrected Wilcoxon signed-rank analysis was used to identify the reason for the difference in the intergroup comparison of differences of PTX-3 IgG positivity using ELISA, and the Mann-Whitney U test was used in the comparison of the mean age of the patients according to sex, and p<0.05 was accepted as statistically significant.

## RESULTS

Of the 36 patients who underwent posterolateral thoracotomy between January 2013 and June 2014, 24 (66.7%) patients were men and 12 (33.3%) were women. The mean age of the men was 43.87±18.64 (range, 18-68) years, and the mean age of the women was 52.33±17.38 (range, 21-80) years. No significant difference was detected between the mean ages of the men and women (p=0.313, p>0.05). Twenty-four patients were aged below 65 years, and 12 patients older than 65 years. Surgery was performed due to benign reasons in 25 patients, whereas it was performed due to malignant reasons in 11 patients. Seven patients who underwent surgery due to malignant reasons were aged over 65 years. The pre-op, and post-op day one, three and seven levels of PTX-3 in patients aged below 65 years were found statistically significantly lower (p<0.05) (Table-I). The pre-op and post-op day one, three and seven levels of WBCs and CRP of patients were found statistically significant higher when compared between themselves, regardless of age range (p<0.05). However, the PTX-3 levels were found statistically insignificant in the comparison between the time points. All changes except the matches on postoperative days one and three in CRP levels were found statistically significant in the Bonferroni-corrected Wilcoxon signed-ranks analysis, which was performed to identify the times from which the differences originated (p<0.0083). The change between preoperative levels of WBCs and post-op levels on days one and three and the change between WBC levels on post-op day 1 and post-op days 3 and 7 were found statistically significant (p<0.0083). The changes in PTX-3 levels between the pre-op levels and post-op days one, three and seven were found statistically insignificant (p>0.05) (Table-II).

**Table-I:** The mean distribution of the PTX-3 levels in accordance with the age groups.

PTX-3	<65 years (n=24)	>65 Years (n=12)	p

	Mean±SD	Mean±SD	
Pre-op. (ng/mL)	4.16±5.06	15.57±14.36	0.001
Post-op. day 1 (ng/mL)	4.63±7.01	17.97±26.49	0.003
Post-op. day 3 (ng/mL)	5.52±10.74	12.81±19.32	0.010
Post-op. day 7 (ng/mL)	7.07±15.19	9.18±7.83	0.030

**Table-II:** The mean distribution of pre-op WBC, CRP, PTX-3 levels and on post-op day 1, day 3, and day 7.

		Mean±SD	Min-Max	p
CRP (µg/mL)	Pre-op	21.78±39.87	1-221	0.001
	Post-op day 1	92.06±56.42	32-221	
	Post-op day 3	77.07±46.7	23.8-188	
	Post-op day 7	44.18±37.29	3-184	
WBC	Pre-op	8.87±2.28	5.3-16.7	0.001
	Post-op day 1	12.52±3.41	7.5-23.1	
	Post-op day 3	9.9±2.55	4.9-17	
	Post-op day 7	8.76±2.28	3.4-13.3	
PTX-3 (ng/mL)	Pre-op	7.97±10.55	0-53	0.069
	Post-op day 1	9.08±17.13	0-97	
	Post-op day 3	7.95±14.33	0-72	
	Post-op day 7	7.74±13.23	0-70	

**Fig.1:**
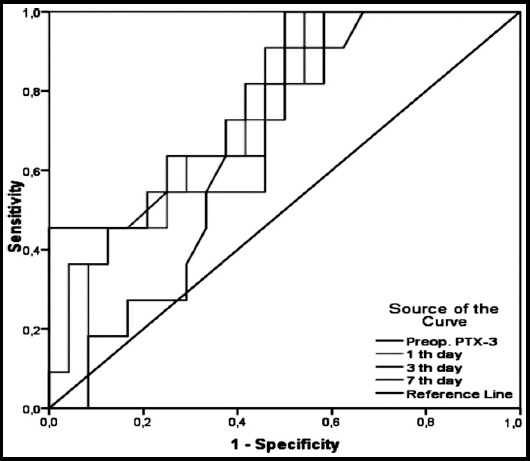
The estimation strength of the malignant patients of PTX-3 with the ROC analysis.

The correlation between the pre-op and post-op days one, three and seven levels of PTX-3 with CRP and WBC levels was found statistically insignificant (p>0.05). Regarding the estimation of strength of PTX-3 in the differentiation of malignant and benign patients, the optimal cut-off levels were 1.37 ng/mL (p=0.013) with pre-op sensitivity and specificity of 45.5% and 100%, respectively; on postop day one, the optimal cut-off levels were 0.53 ng/mL with sensitivity and specificity of 100% and 45.8%, respectively (p=0.030); and on post-op day three, optimal cut-off levels were found as 0.46 ng/mL, with sensitivity and specificity of 100% and 50%, respectively (p=0.012) (Table-III). The area under the curve (AUC) for PTX-3 levels in the receiver operating characteristics (ROC) analysis was found statistically significant (p<0.05) (Fig.1). The pre-op and post-op day 3 PTX-3 levels of the malignant patients were found statistically significantly higher compared with the pre-op and post-op day 3 PTX-3 levels of the benign patients (p<0.05). No statistically significant difference was detected regarding the other variables between the groups (p>0.05) (Table-IV).

**Table-III:** PTX-3 levels of the malignant and benign patients with ROC analysis results performed for estimation strength of the malignant cases.

	PTX-3	Area	P	Confidence Interval (95%)	Optimal Cut-off	Sensitivity	Specificity
Pre-op. (ng/mL)	0.765	0.013	0.594	0.936	1.37	45.5	100.0
Post-op. day 1	0.731	0.030	0.564	0.898	0.53	100.0	45.8
Post-op. day 3	0.767	0.012	0.609	0.925	0.46	100.0	50.0
Post-op. day 7	0.655	0.145	0.476	0.834	1.62	90.9	50.0

**Table-IV:** The mean distribution of the PTX-3 levels in malignant and benign patients.

PTX-3		Mean±SD	Min.-Max.	p*
Pre-op. (ng/mL)	Benign	6.39±10.85	0-28	0.026
	Malignant	11.56±9.30	0.65-53	
	Total	7.97±10.55	0-53	
Post-op. day 1	Benign	9.07±20.22	0-18	0.055
	Malignant	9.10±6.82	65-97	
	Total	9.08±17.13	0-97	
Post-op. day 3	Benign	6.93±15.54	0-40	0.024
	Malignant	10.28±11.43	62-72	
	Total	7.95±14.33	0-72	
Post-op. day 7	Benign	7.47±15.15	0-29	0.144
	Malignant	8.32±8.19	54-70	
	Total	7.74±13.23	0-70	

* Mann-Whitney U analysis

## DISCUSSION

Pentraxin-3 is a member of the pentraxin superfamily, which includes CRP, involved in innate immune responses and inflammation. PTX-3 differs from CRP in several aspects: gene organization, structure, inducing stimuli, and recognized ligands.^12^,^13^ PTX-3 plays an important role in innate immunity, inflammation, vascular integrity, fertility, pregnancy, and also in the central nervous system.^14^ In our study, we aimed to evaluate the body immune response due to posterolateral thoracotomy, and inflammation levels. Therefore, we studied the preoperative PTX-3, CRP, and WBC levels in the serums of patients, and on post-op. days one, three and seven. The pre-op. and post-op. days one, three and seven levels of WBCs and CRP were statistically significant in the comparison between themselves, however the comparison of PTX-3 levels was found statistically insignificant. Tatli et al. reported that PTX-3 levels significantly increased in patients with pulmonary contusion compared with healthy controls, and stated that PTX-3 might be used as a marker in the identification of the pulmonary contusion.^15^ Although PTX-3 has been used as a marker of inflammation and immune response in the last 15 years, the pre-op and post-op days one, three and seven levels were found statistically insignificant in our study. However, it has been reported that the new PTX-3 biomarker was significantly increased in inflammation due to lung cancer, and in inflammatory diseases.^16^-^19^ In our study, posterolateral thoracotomy was performed in 11 patients due to lung cancer, intrapericardial pneumonectomy in two patients, sleeve lobectomy in one patient, bilobectomy in one patient, and lobectomy in seven patients; lymph node dissection was performed in all patients. Postoperative epidermoid carcinoma was reported in six patients, adenocarcinoma in four patients, and carcinoid tumor was reported in one patient. In compliance with the previous studies, the pre-op. and post-op. day 3 PTX-3 levels of patients with lung cancer were found statistically significantly higher compared with the pre-op. and post-op. day three PTX-3 levels of benign patients in our study.^16^,^19^-^21^ The AUC levels in the ROC analysis conducted for the differentiation of malignant and benign cases were found statistically significant for the estimation strength of PTX-3 for pre-op. optimal cut-off levels 1.37 ng/mL (p=0.013), post-op day 1 0.53 ng/mL (p= 0.030), and day 3 0.46 ng/mL (p=0.012). Infante et al. reported that there was an association between blood PTX-3 levels and the development of lung cancer.^21^ Although our patient numbers were small, we also suggest that there might be an association between lung cancer and PTX-3 levels. Although no studies have been conducted on this topic, we found that PTX-3 was significantly higher in patients aged over 65 years compared with patients younger than 65 years; PTX-3 might be a marker of inflammation and of change in the body organs due to aging.

CRP is an acute inflammatory protein that is known to increase in inflammation and infections, and is primarily produced in the liver, smooth muscle cells, macrophages, endothelial cells, lymphocytes, and adipocytes.^22^ Plasma CRP levels may increase from 1 µg/mL to 500 µg/mL within 24- 72 hours in cases of inflammation, infection, trauma or cancer, which cause serious tissue damage.^23^,^24^ We showed that levels of WBCs and CRP increased due to posterolateral thoracotomy on post-op. days 1, 3, and 7 compared with preoperative levels, and the increase was found statistically significant in the within group comparison. The increase in the CRP and WBC levels continued on postoperative days 1 and 3, and started to decrease after day 3. Previous studies have reported that CRP levels increased in cardiovascular and vascular diseases, in infectious diseases such as cholecystitis, pancreatitis, appendicitis, meningitis, pneumonia, and in trauma and inflammation.^24^,^25^ We also detected a statistically significant increase in CRP and WBC levels as an immune response due to posterelateral thoracotomy, independent of an infectious pathology. It should be known that these increased levels were not an indicator of infection in the early period, and was an immune response of the body; however, the maintenance of increase and/or detection of no decrease in CRP and WBC levels after day 3, and detection of symptoms of fever in clinical follow-up may suggest an infection independent of the immune response to poster lateral thoracotomy.

## CONCLUSION

It should be born in mind that PTX-3 levels are statistically significantly increased in patients with lung cancer and/or aged over 65 years, and the correlation between the PTX-3, CRP, and WBC levels in patients who underwent posterolateral thoracotomy was insignificant. WBC and CRP levels were significantly increased due to the posterolateral thoracotomy, and the increase might continue until post-op. day 3 without any symptoms of infection; however, the detection of an increase and/or the tendency for no decrease might be an indicator of infection. We suggest that the novel marker PTX-3 might be an indicator of the inflammatory response in patients to posterolateral thoracotomy with lung cancer and/or aged over 65 years, and might be used in the diagnosis and follow-up of the prognosis of patients with lung cancer. Further studies might be instructive for researchers.

### Author Contribution

**ID, AAG:** Conceived, designed and did statistical analysis & editing of manuscript.

**ID, AAG, IG, SK:** Did data collection and prepared the manuscript.

**ID:** Did review and final approval of manuscript.
